# Methyldigoxin-Induced Visual Hallucinations in an Elderly Patient with Advanced Fazekas Scale 3 Leukoaraiosis: A Diagnostic Challenge and Case Report

**DOI:** 10.1159/crn/abyag018

**Published:** 2026-10-09

**Authors:** Mario Ćurković, Tamer Salha, Mladen Oršolić, Nika Ćurković

**Affiliations:** a Department of Family Medicine, Osijek-Baranja County Health Center, Osijek, Croatia; b Department of Family Medicine, Faculty of Medicine, Josip Juraj Strossmayer University of Osijek, Osijek, Croatia; c Department of Radiology, Osijek-Baranja County Health Center, Osijek, Croatia; d Department of Radiology, Faculty of Medicine, Josip Juraj Strossmayer University of Osijek, Osijek, Croatia; e Department of Radiology, Faculty of Dental Medicine and Health Osijek, Osijek, Croatia; f Department of Family Medicine, Health Center of Vukovar-Srijem County, Županja, Croatia; g Department of Family Medicine, Health Center Zagreb - Centar, Zagreb, Croatia

**Keywords:** Case report, Methyldigoxin, Visual hallucinations, Leukoaraiosis, Atrial fibrillation

## Abstract

**Introduction:**

Digoxin and its derivatives remain valuable adjunctive therapies for rate control in atrial fibrillation, yet their narrow therapeutic index poses a significant risk for neurotoxicity, particularly in the geriatric population.

**Case presentation:**

We report the case of a 79-year-old female with heart failure and chronic kidney disease who developed vivid, complex visual hallucinations 2 weeks after initiating methyldigoxin therapy. The patient’s medical history and subsequent magnetic resonance imaging revealed advanced subcortical vascular encephalopathy, characterized by confluent perivascular gliosis (Fazekas scale 3). This structural background initially raised suspicion of a transient ischemic attack or progressing vascular dementia. However, the patient maintained a high cognitive baseline (MMSE 28/30), and the hallucinations – which included perceiving her deceased husband and grandson – resolved completely within 4 days of methyldigoxin discontinuation. This rapid recovery, combined with the absence of new focal neurological deficits, suggests digitalis induced neurotoxicity rather than a primary cerebrovascular event.

**Conclusion:**

This case underscores the “clinical-radiological discrepancy” often encountered in elderly patients, where advanced neuroimaging findings may act as a confounding factor. It highlights the necessity of maintaining a high index of suspicion for drug-induced causes of acute neurological symptoms in patients with a “vulnerable brain” due to severe leukoaraiosis. Careful monitoring of digitalis compounds in patients with impaired renal function is mandatory to prevent misdiagnosis and unnecessary interventions.

## Introduction

Digoxin and its derivatives, such as methyldigoxin, have a long history of clinical use in cardiology. While no longer considered first-line therapy, they remain valuable adjunctive options for heart failure with reduced ejection fraction and rate control in atrial fibrillation [1, 2]. Despite their benefits, toxicity remains a persistent concern, occurring in up to 20% of patients [3]. While gastrointestinal and cardiac manifestations of digoxin toxicity are well established, central nervous system effects – including confusion, delirium, and hallucinations – are also well described, particularly in older adults, in whom they may represent the predominant or even sole manifestation of toxicity and are, therefore, sometimes misattributed to aging, dementia, or primary neurological disease [4–6]. This diagnostic distinction becomes particularly challenging in patients with pre-existing structural brain changes, such as advanced leukoaraiosis. This case report was prepared in accordance with the CARE guidelines, and the completed checklist is provided as supplementary material.

## Case Presentation

A 79-year-old woman with a history of heart failure with reduced ejection fraction (New York Heart Association [NYHA] Functional Class III), atrial fibrillation, and chronic kidney disease presented to her primary care physician with acute-onset visual hallucinations. Her medical history included breast cancer (treated with surgery and radiochemotherapy in 2005), presbyopia, and chronic gastritis. Her baseline therapy consisted of bisoprolol, sacubitril/valsartan, empagliflozin, eplerenone, and dabigatran.

Due to persistent tachyarrhythmia (heart rate [HR] 139 bpm) and borderline hypotension, methyldigoxin was introduced (loading dose: 1 tablet three times daily for 3 days; maintenance: 1 tablet twice daily). Two weeks after initiating therapy, the patient developed complex, vivid visual hallucinations. She described seeing and conversing with her deceased husband and perceiving her grandson’s presence in the room. These episodes occurred frequently when the patient was idle and typically vanished upon direct ocular engagement.

### Neurological and Diagnostic Evaluation

Upon neurological evaluation, the patient was oriented with a Mini-Mental State Examination (MMSE) score of 28/30. No focal motor or sensory deficits, nystagmus, or visual field defects were noted. Laboratory findings showed a creatinine level of 89 µmol/L (eGFR 54 mL/min/1.73 m^2^, KDIGO G3aA1) and normal electrolytes. Serum digoxin levels, measured after the onset of symptoms and subsequent discontinuation, were <0.30 nmol/L. Additionally, broader laboratory evaluations (including complete blood count, C-reactive protein, extended metabolic panel, and thyroid function) excluded systemic infections, acute metabolic derangements, or thyroid dysfunction as potential triggers for the hallucinations. Best-corrected visual acuity was preserved (20/30 in both eyes with corrective lenses), ruling out significant sensory deprivation as a primary cause.

### Neuroimaging

Brain magnetic resonance imaging (MRI) confirmed pronounced multi-infarct microangiopathy of the white matter, characterized by confluent perivascular gliosis in the deep and subcortical white matter (Fazekas scale 3). Periventricular zones showed chronic hypoperfusion (Fig. 1). Additional MRI sequences (Fig. 2) confirmed no signs of acute ischemia on DWI and ADC maps (Fig. 2a, b), absence of microhemorrhages on T2* GRE (Fig. 2c), and prominent multicentric, partly confluent perivascular hyperintensities on FLAIR (Fig. 2d).

**Fig. 1.**
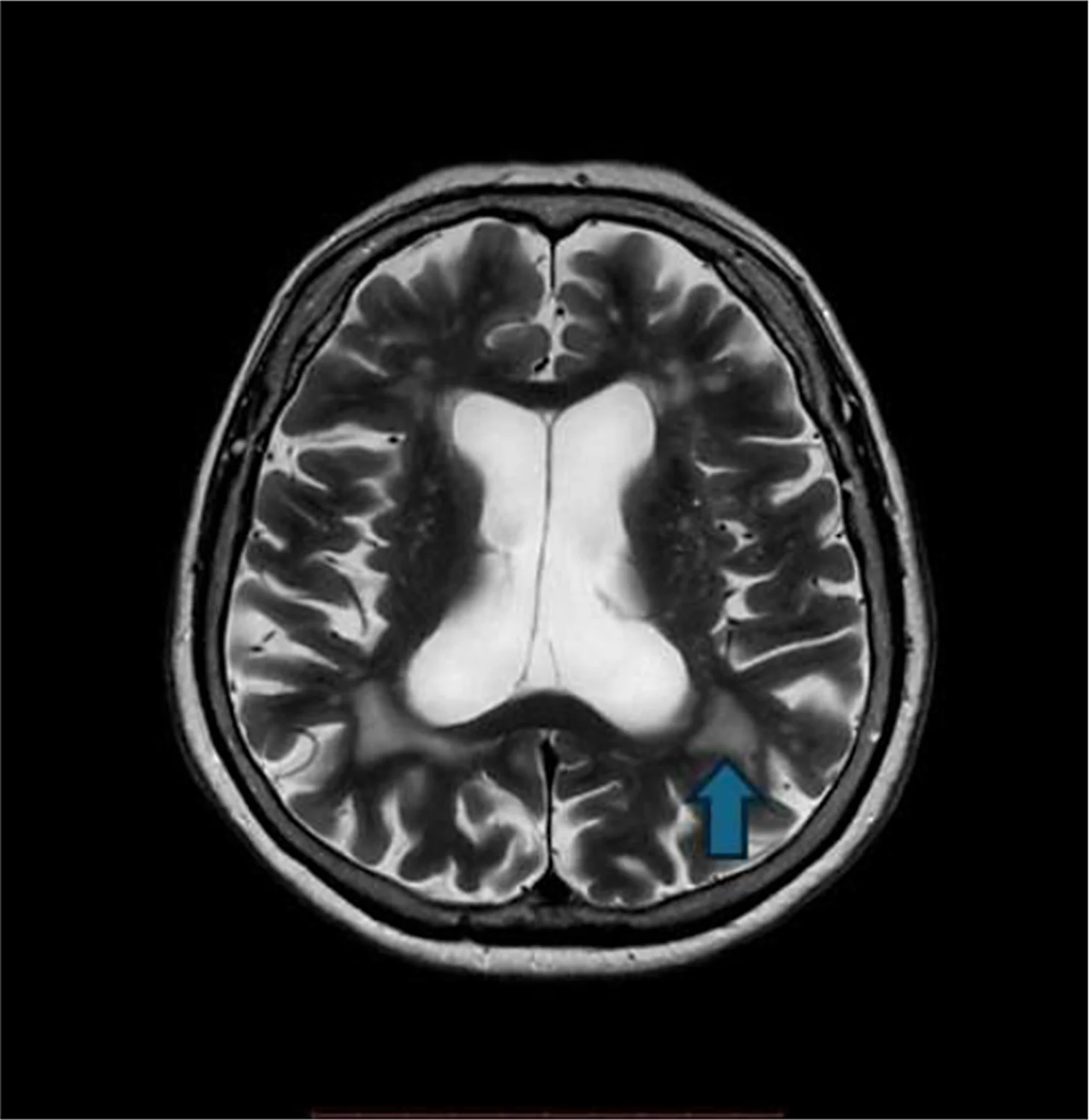
T2-weighted MRI – transverse section through the cerebral hemispheres showing multiple areas of white matter infarct microangiopathy (arrow highlights one area).

**Fig. 2.**
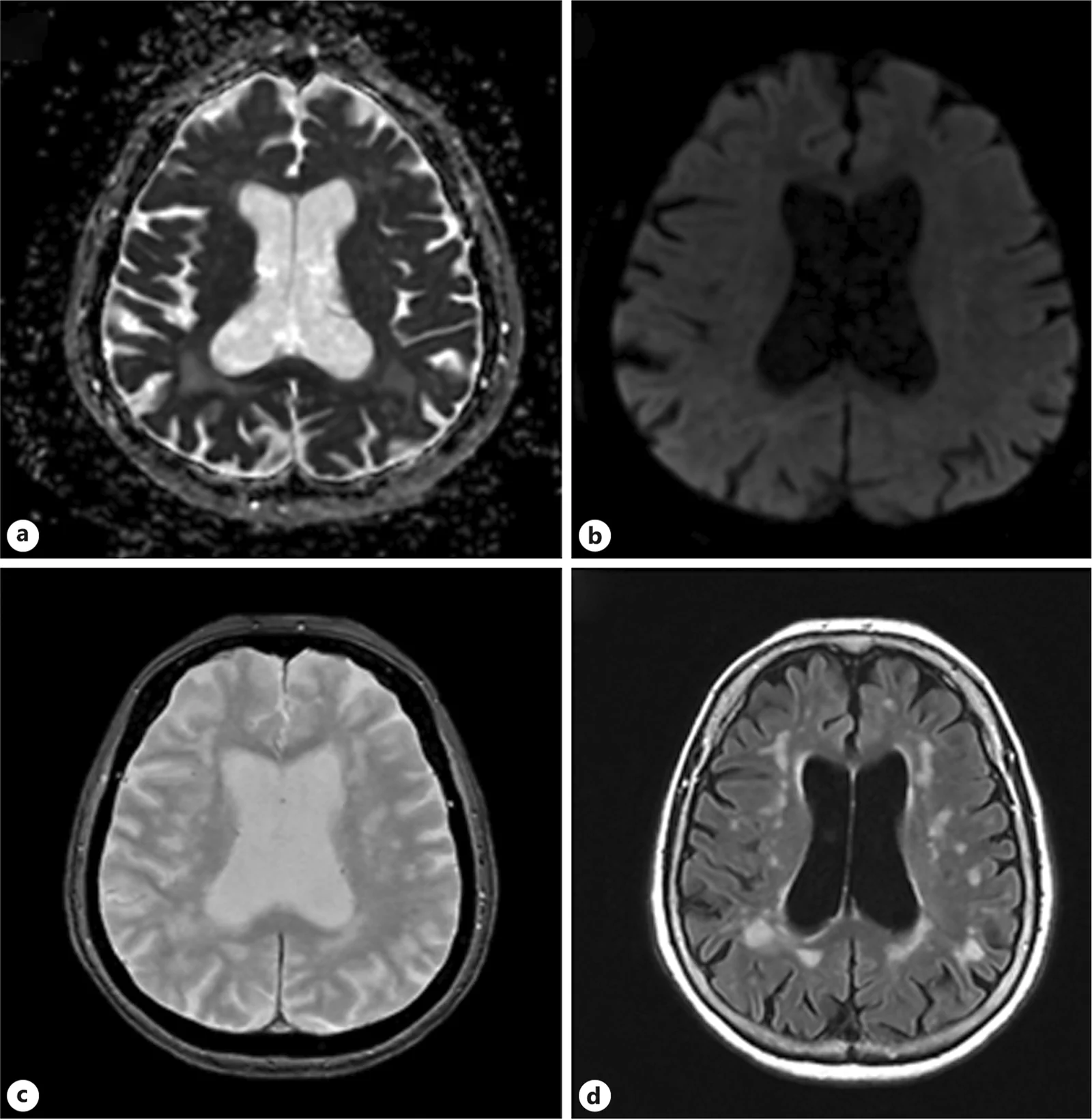
Additional brain MRI sequences for the assessment of cerebral small vessel disease (CSVD) severity and exclusion of acute ischemia. Axial sections through the cerebral hemispheres and lateral ventricles. **a**, **b** ADC map and DWI 1000 showing no signs of acute ischemia (no restriction of diffusion on diffusion-weighted sequences). **c** T2* GRE showing no signs of microhemorrhage (no signal dropout on T2*). **d** FLAIR showing characteristic multicentric, partly confluent perivascular (periventricular) white matter hyperintensities, typical of advanced CSVD.

### Intervention and Outcome

Given the temporal correlation between drug initiation and symptom onset, methyldigoxin was immediately discontinued. The visual hallucinations resolved completely within 4 days.

## Discussion

The diagnostic challenge in this case lies in the “clinical-radiological discrepancy.” While the MRI showing advanced small vessel disease (Fig. 1; 2) provided a clear substrate for vascular dementia or transient ischemic attacks, the patient’s stable cognitive baseline and the specific nature of the hallucinations pointed toward a pharmacological etiology. Neuropsychiatric manifestations of digitalis toxicity can occur even at “therapeutic” serum concentrations, especially in elderly patients with impaired renal function [5, 6]. A key limitation is that serum digoxin levels (<0.30 nmol/L) were measured after drug discontinuation. Consequently, this value reflects post-cessation clearance and cannot definitively confirm or exclude supratherapeutic levels at symptom onset. Furthermore, other etiologies of visual hallucinations in the elderly – such as dementia with Lewy bodies, Parkinson’s disease dementia, Charles Bonnet syndrome, and occipital lobe seizures – were considered less likely due to the absence of parkinsonism, REM sleep behavior disorder, severe visual loss, or paroxysmal EEG abnormalities, as well as the complete resolution upon drug withdrawal. The neurotoxic mechanism of digitalis is thought to involve the inhibition of neuronal Na+/K+ ATPase, leading to altered membrane potentials, disturbed neurotransmitter release (e.g., disruption of visual pathways), and altered central cholinergic transmission [7, 8].

To objectively assess the causality in this case, the Naranjo adverse drug reaction probability scale was applied [9]. The case yielded a score of 3, indicating a “possible” adverse drug reaction. This score reflects the complex diagnostic landscape: while the *dechallenge* was successful, the significant background of white matter microangiopathy (Fazekas 3) must be acknowledged as a potential confounding factor.

The temporal relationship – symptoms appearing after initiation and vanishing 4 days post-discontinuation – is the strongest evidence for methyldigoxin-induced neurotoxicity (Fig. 3). We hypothesize that the advanced leukoaraiosis may have lowered the patient’s “neurobiological threshold,” making her brain more vulnerable to pharmacological interference. In this context, the MRI served more as a tool to rule out acute ischemia and a prognostic indicator of brain vulnerability.

**Fig. 3.**
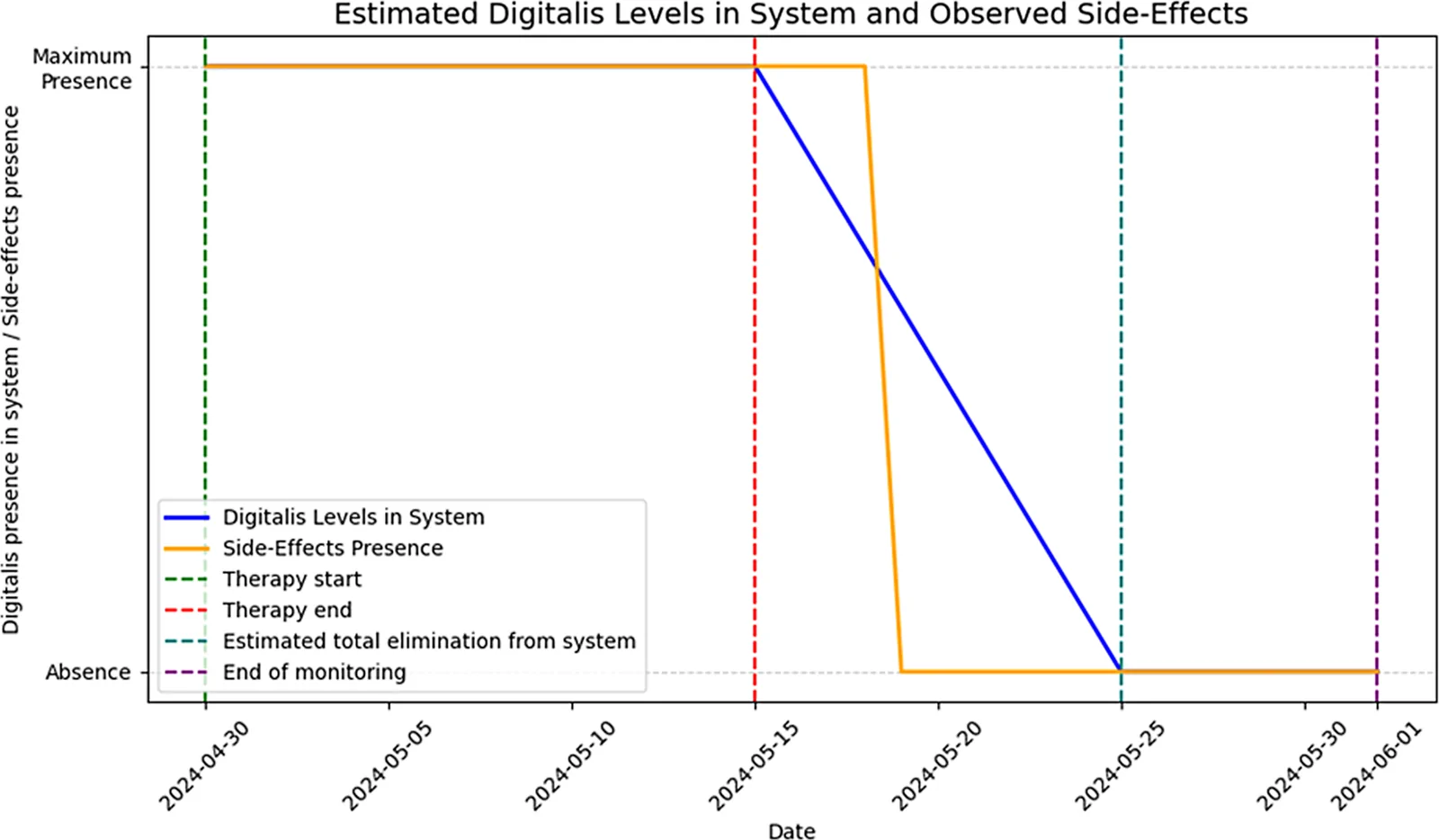
The plot illustrates the relationship between digitalis levels in the system, the presence of side effects, and key timeline markers for therapy and monitoring.

## Conclusion

In geriatric patients, new-onset neurological or psychiatric symptoms should be screened for drug-induced causes before being attributed to irreversible vascular decline. This case underscores the importance of vigilance when using digitalis compounds in patients with documented microangiopathy and chronic kidney disease.

## Acknowledgments

During the preparation of this manuscript, the authors used a Large Language Model solely to improve the language clarity and readability of the text. The authors reviewed and edited the output and take full responsibility for the content and integrity of the publication.

## Statement of Ethics

This study protocol was reviewed and approved by the Ethics Committee of the Health Center Osijek Baranja County, Approval No. 03-4739/2024. Written informed consent was obtained from the patient for publication of the details of their medical case and any accompanying images. The CARE Checklist for this case report is available as online supplementary material (for all online suppl. material, see https://doi.org/10.1159/crn/abyag018).

## Conflict of Interest Statement

The authors have no conflicts of interest to declare.

## Funding Sources

The authors received no financial support for the research, authorship, and/or publication of this article.

## Author Contributions

Mario Ćurković: conceptualization, writing – original draft, project administration, and supervision. Tamer Salha: investigation, data curation, and resources. Mladen Oršolić: investigation and validation. Nika Ćurković: formal analysis and writing – review and editing. All authors have read and approved the final manuscript.

## Data Availability

The data that support the findings of this study are not publicly available due to privacy reasons but are available from the corresponding author upon reasonable request.
